# Gel immersion endoscopy for evaluation of a gastric submucosal tumor during endoscopic ultrasound-guided fine-needle aspiration

**DOI:** 10.1055/a-2362-0875

**Published:** 2024-08-07

**Authors:** Koichi Soga, Takeshi Fujiwara, Fuki Hayakawa, Ryosuke Oura, Ikuhiro Kobori, Masaya Tamano

**Affiliations:** 1 26263 Gastroenterology, Dokkyo Medical University Saitama Medical Center, Koshigaya, Japan

Gel immersion is a well-known and useful method for performing endoscopic procedures. Viscoclear gel (Otsuka Pharmaceutical Factory, Tokushima, Japan) is used to secure the visual field and prevent rapid runoff through the appropriate viscosity 1 . After injection of the clear gel, instead of water, into the gastrointestinal lumen, the visual field can be secured within the space occupied by the gel. We present the evaluation and diagnosis of a gastric submucosal tumor using endoscopic ultrasound (EUS) and the gel immersion method. We considered that, unlike water, the gel helped effectively visualize the gastric submucosal tumor on EUS ( Video 1 ).

Gel immersion endoscopy is used to evaluate a gastric submucosal tumor during endoscopic ultrasound-guided fine-needle aspiration.Video 1

A 76-year-old woman was admitted to the endoscopy unit for further examination of an enlarged gastric submucosal tumor on the fornix ( Fig. 1 ). We attempted first to evaluate the submucosal tumor on EUS by filling the lumen with water; however, owing to its low viscosity, the required volume of water could not be maintained in the target area. Moreover, we realized the difficulty of intervention on the submucosal tumor in a patient with coughing associated with the water reflux. Therefore, we switched to gel immersion EUS instead. The gel was injected through the working channel until the gastric wall was adequately extended on EUS. The gel remained in the target area to facilitate observation ( Fig. 2 **a** ). Furthermore, because the submucosal tumor protruded into the lumen, it floated like an elevated polyp, making it easy to evaluate, with a clear view, and allowing safe EUS-guided fine-needle aspiration to be performed ( Fig. 2 **b,c** ).

**Fig. 1:**
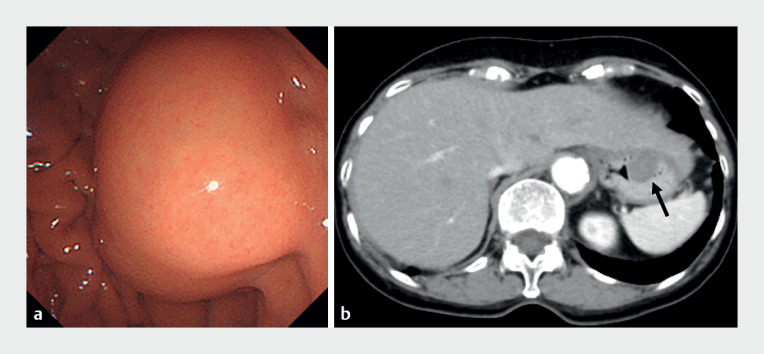
Imaging at our hospital confirming the presence of the gastric submucosal tumor on the posterior wall of the fornix on: **a** esophagogastroduodenoscopy; **b** abdominal computed tomography.

**Fig. 2:**
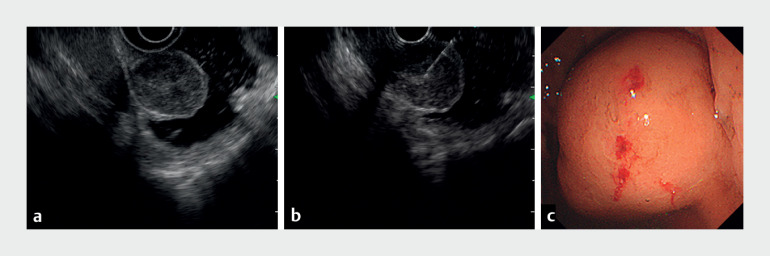
Images during the gel immersion endoscopic ultrasound (EUS)-guided fine-needle aspiration (EUS-FNA) procedure showing: **a** clear observation of the gastric submucosal tumor on EUS after the injection of 180 mL of gel through the working channel to adequately distend the gastric wall; **b** EUS-FNA being safely performed on the tumor, which has floated into the lumen like an elevated polyp, making it easy to evaluate; **c** endoscopic view of the tumor after the performance of gel immersion EUS-FNA without complications.

Gel immersion EUS provides better observation of gastric submucosal tumors, similarly to the underwater technique 2 . Gel immersion procedures create lower levels of intraluminal pressure and allow for better maintenance of wall tension compared with air or water immersion. We also considered that management of the submucosal tumor using gel immersion EUS was less stressful for both patient and physicians. This case indicates that the gel immersion technique is helpful for diagnosis and intervention for submucosal tumors during EUS-related procedures.

Endoscopy_UCTN_Code_TTT_1AO_2AG_3AD

## Notes

Endoscopy E-Videos https://eref.thieme.de/e-videos
*E-Videos* is an open access online section of the journal *Endoscopy* , reporting on interesting cases and new techniques in gastroenterological endoscopy. All papers include a high-quality video and are published with a Creative Commons CC-BY license. Endoscopy E-Videos qualify for HINARI discounts and waivers and eligibility is automatically checked during the submission process. We grant 100% waivers to articles whose corresponding authors are based in Group A countries and 50% waivers to those who are based in Group B countries as classified by Research4Life (see: https://www.research4life.org/access/eligibility/ ). This section has its own submission website at https://mc.manuscriptcentral.com/e-videos .
